# Impact of post-resection maxillofacial defects on outcomes of 3D-printed prosthetic rehabilitation in oral cancer patients: a scoping review protocol

**DOI:** 10.1038/s41405-026-00476-3

**Published:** 2026-08-14

**Authors:** Smitha Sammith Shetty, Shibani Shetty, Sameep S. Shetty, Swapna BV

**Affiliations:** 1 Department of Oral and Maxillofacial Pathology and Microbiology, Manipal College of Dental Sciences, Manipal Academy of Higher Education, Manipal, India; 2 Department of Oral Pathology and Microbiology, M.R. Ambedkar Dental College & Hospital, Bangalore, India; 3 Department of Oral and Maxillofacial Surgery, Manipal College of Dental Sciences, Mangalore, Manipal Academy of Higher Education, Manipal, India; 4 Department of Prosthodontics and Crown and Bridge, Manipal College of Dental Sciences, Manipal Academy of Higher Education, Manipal, India

**Keywords:** Prosthetic dentistry, Oral cancer

## Abstract

**Introduction:**

Oral cancer patients undergoing surgical resection often develop extensive maxillofacial defects that compromise function, aesthetics, and quality of life. Three-dimensional (3D) printing has enabled the development of customized, patient-specific prostheses, offering improved precision in rehabilitation. However, the extent to which defect type and location influence prosthetic outcomes remains inadequately synthesized in the current literature.

**Objective:**

To map and synthesize the available evidence on the impact of type of maxillofacial defect on outcomes of 3D-printed prostheses in oral cancer patients.

**Inclusion criteria:**

This review will include studies involving oral cancer patients with surgically acquired maxillofacial defects rehabilitated using a 3D-printed prosthesis. Studies involving intraoral and extraoral defects that report functional, clinical, prosthetic, or aesthetic outcomes of 3D-printed prostheses will be included.

**Methods:**

A comprehensive literature search will be conducted across databases including PubMed, Scopus, Web of Science, Embase, and Cochrane Library using relevant keywords. Two independent reviewers will perform study selection through title/abstract screening followed by full-text review. Data will be extracted using a structured template recording the study characteristics, surgical defect characteristics, prosthetic rehabilitation details, and outcome measures. Findings will be synthesized descriptively and presented in tabular and narrative formats to identify the research gaps. The review will be conducted and reported in accordance with the PRISMA-ScR guidelines.

**Review registration:** Open Science Framework https://doi.org/10.17605/OSF.IO/C8STU

## Introduction

Oral cancer constitutes a significant global health concern, with an estimated 758,000 individuals diagnosed with cancers of the lip, oral cavity, and pharynx (LOCP), posing a considerable public health challenge worldwide (Global Cancer Observatory, 2022) [1]. Oral cancer requires aggressive surgical management that results in substantial loss of anatomical structures and tissues [2]. These resections frequently lead to complex post-resection maxillofacial defects involving the maxilla, mandible, palate, and associated facial structures. Such defects affect essential functions, including speech, mastication, deglutition, and facial aesthetics, thereby significantly compromising the patient’s a patient’s self-confidence, social well-being, and overall quality of life. The rehabilitation of these defects is not merely restorative but also rehabilitative in nature, aiming to reinstate both oral function and psychosocial well-being of the patient [2, 3].

The nature of post-resection maxillofacial defects is inherently complex and heterogeneous. These defects vary widely in size, anatomical location, and extent of tissue involvement, which in turn directly influences prosthetic rehabilitation outcomes [4, 5]. Various classification systems define and categorize these defects such as Aramany’s classification for the partially edentulous maxillectomy defects, Brown classification addresses defects that encompass both vertical and horizontal dimensions, as well as the palatal aspect following maxillectomy, Cordeiro classification for palatomaxillary defects, and the Cantor-Curtis and Jewer-Boyd (HCL) systems for mandibular continuity defects [6, 7]. These defect classification systems are critical in prosthetic rehabilitation, as they enable consistent correlation between defect characteristics and treatment planning, prosthesis design, predict functional outcomes and compare success rates across clinical research [7].

Restoring large facial defects is prosthetically difficult due to lack of anatomic undercuts, inadequate retention, mobility of soft tissues, and in addition the weight of prosthesis can compromise the stability [4, 8]. Additionally, disruption of anatomical continuity often leads to functional impairments such as compromised mastication, and mandibular deviation [9]. These challenges are further compounded by post-treatment sequelae such as fibrosis, trismus, and fibrosis of masticatory muscles [10].

Traditionally, maxillofacial prosthetic rehabilitation has relied on conventional fabrication techniques involving manual impressions and laboratory processing. While these approaches have limitation pertaining to their ability to accurately replicate complex anatomical defects and involve multiple time-consuming steps which can be distressing for patients [5]. The advent of three-dimensional (3D) printing, also referred as rapid prototyping (RP) or additive manufacturing, has been proposed as a shift in the field of maxillofacial prosthodontics [11]. This technology facilitates the production of highly precise, patient-specific prosthesis through the integration of 3D scanning and computer-aided design (CAD) [12]. However, the success of this technology is not solely a function of the printing process itself but is also shaped by the characteristics of the defect being restored.

Despite these technological advancements, the outcomes of 3D-printed prosthetic rehabilitation are not uniform across all cases. The extent and anatomic location of the defect usually influence the degree of functional impairment and difficulty in prosthetic rehabilitation. These characteristics play a critical role in determining the success of prosthetic intervention [13]. The maxillary defects involving the palate primarily affect speech, nasal resonance and swallowing, necessitating customized obturator designs that achieve effective oro-nasal separation [14]. Similarly the mandibular defects often lead to deviation and occlusal discrepancies, requiring prosthetic strategies that restore mandibular continuity and function [15]. The restoration of large both intraoral and extraoral maxillofacial defects is challenging due to the limited retention and enhanced expectations to achieve optimal aesthetic and functional rehabilitation [16].

Although the use of 3D printing in maxillofacial rehabilitation has been widely explored, the available evidence examining the relationship between post-resection maxillofacial defects and prosthetic outcomes remains limited. Therefore, this scoping review aims to systematically map and synthesize the available literature on the impact of post-resection maxillofacial defects on the outcomes of 3D-printed prosthetic rehabilitation in oral cancer patients. By identifying patterns, trends, and gaps in the current evidence, this review seeks to provide valuable insights that can guide clinicians in the field of maxillofacial prosthodontics. This review aligns with the United Nations Sustainable Development Goals [17], particularly SDG 3 (Good Health and Well-being), by addressing rehabilitation and quality of life in oral cancer patients, and SDG 9 (Industry, Innovation and Infrastructure), through the exploration of emerging 3D printing technologies in maxillofacial prosthetics.

### Objective

To map and synthesize the available evidence on the impact of the type of maxillofacial defect on outcomes of 3D-printed prostheses in oral cancer patients.

## Review questions

1. What is the impact of different types of post-resection maxillofacial defects on outcomes of 3D-printed prosthetic rehabilitation in oral cancer patients?

- How do variations in maxillofacial defect classification (e.g., maxillary vs. mandibular, extent and location of resection) influence the functional and clinical outcomes (mastication, speech, prosthesis retention and stability) of 3D-printed prosthetic rehabilitation in oral cancer patients?
- How do variations in maxillofacial defect classification influence the aesthetic and patient-reported outcomes (quality of life, satisfaction, psychosocial well-being) of 3D-printed prosthetic rehabilitation in oral cancer patients?

## Methodology

This scoping review will be conducted in accordance with the Joanna Briggs Institute (JBI) methodological framework [18] for scoping reviews, with reporting structured according to the PRISMA-ScR (Preferred Reporting Items for Systematic Reviews and Meta-Analyses extension for Scoping Reviews) checklist [19].

## Eligibility Criteria (PCC Framework)

This scoping review will apply the Population–Concept–Context (PCC) framework to address the review questions on the impact of the type of maxillofacial defect on outcomes of 3D-printed prostheses in oral cancer patients.

### Population

The population of interest comprises patients with intraoral and/or extraoral maxillofacial defects acquired following treatment for oral cancer, classified under any defect classification system. Experimental, observational, and descriptive studies relating to patients with maxillofacial defects acquired due to oral cancer resection will be included; studies involving defects arising from causes other than oral cancer will be excluded.

### Concept

This review will explore studies that have adopted 3D-printing technology for prosthetic rehabilitation in oral cancer patients. Studies focusing on prosthetic rehabilitation using traditional (non-3D-printed) methods will not be considered.

### Context

Evidence from any clinical or translational research will be considered, without geographic restriction.

## Types of sources

This scoping review will consider evidence sources that are in line with the inclusion criteria, that includes the randomized and non-randomized controlled trials, observational studies that include cohort, case-control, and cross-sectional studies, and also descriptive studies such as case series and case reports. In addition, systematic reviews and meta-analyses will be screened for studies related to 3D printing and maxillofacial rehabilitation in oral cancer patients. Editorials, opinion articles, and studies not involving human subjects will be excluded.

## Search strategy

The search strategy will aim to identify both published and unpublished (grey literature) studies. A search strategy will include three stages. First, an initial search will be conducted in PubMed and Scopus to identify relevant articles on 3D-printed prosthetic rehabilitation done in oral cancer patients. The index terms and text words in the titles and abstracts of these articles will be further used as keywords to develop a comprehensive search strategy. The final search strategy will include all keywords and index terms and will be used for each database, including PubMed, Scopus, Web of Science, Embase, Cochrane Library, and Google Scholar for grey literature. (Appendix I) The reference lists of all included studies and relevant systematic reviews and meta-analyses will also be screened to identify additional articles.

Articles published only in English and from inception to July 2026 will be considered for comprehensive coverage of evidence. Before implementation, the search strategy will be reviewed by an experienced librarian to ensure its accuracy and comprehensiveness.

## Study/Source of evidence selection

After completing the search, all identified citations will be gathered and uploaded into Rayyan software for further screening (Cambridge, MA 02142 USA) [20] and duplicates will be removed. Before commencing formal screening, the reviewers will undertake a calibration exercise by independently screening a pilot sample of approximately 20–30 records (or 10% of retrieved records, whichever is smaller) using the predefined eligibility criteria. Any discrepancies will be discussed, and the screening criteria will be refined. After pilot testing the screening process, two reviewers (SSaS and SBV) will independently screen the titles followed by the abstracts, using the predefined inclusion criteria. The full text of potentially relevant articles will be imported into the Rayyan software for further screening. (Cambridge, MA 02142 USA) [20]. The full texts of selected studies will be carefully evaluated against the inclusion criteria by two independent reviewers (SSaS and SBV). Inter-reviewer agreement will be evaluated using Cohen’s kappa (κ) statistic. Disagreements between reviewers at any stage of the selection process will be resolved through discussion, and if needed, by consulting a third reviewer (SS). Any studies that do not meet the criteria at this stage will be excluded, with reasons clearly recorded and reported in the final scoping review. The results of the search and study selection process will be comprehensively reported in the final review using a PRISMA 2020 flow diagram [21].

## Data extraction

The data from the final articles included in the scoping review will be extracted using a data extraction tool in the Rayyan software [20]. Data extraction will be performed using a standardized data extraction form, with a focus on study characteristics, surgical defect characteristics, prosthetic rehabilitation details, and outcome measures. Study characteristics will include the author, year, journal, country of the study/case reported, and sample size. Surgical defect characteristics will include tumour/oral cancer site, anatomical site, defect classification used, and type and extent of defect. For studies that describe defects narratively without using a standardized classification but provide sufficient anatomical detail, two independent reviewers (SSS and SBV) will assign the closest corresponding classification based on predefined criteria relevant to the anatomical site. Prosthetic rehabilitation details will include type of prosthesis, 3D-printing fabrication workflow, design and software used, and follow-up data. Outcome measures will be grouped into functional outcomes (e.g., speech intelligibility, mastication, swallowing), prosthesis-related outcomes (e.g., retention, stability, fit, complications), aesthetic outcomes, and patient-reported outcomes (e.g., satisfaction, oral health-related quality of life), with the assessment instrument/method used recorded for each **(**Appendix II**)**. No restrictions will be placed on the outcome measurement instruments used by the included studies. Results will be reported using descriptive summaries, tables, and charts. Any methodological deviations from the protocol will be documented in the final report. This data extraction sheet will be revised and updated as needed during the data extraction process for each included study, and any modifications will be clearly described in the final scoping review.

## Data analysis and presentation

Data extracted from included studies will be analyzed and presented to directly address the review objectives and research questions. Studies will be grouped by anatomical site (e.g., maxillary, mandibular, intraoral, extraoral, or composite defects) and, where reported, by defect classification and extent of resection. Rehabilitation outcomes, including functional, aesthetic, prosthesis-related, and patient-reported outcomes, will be mapped across these defect categories to identify patterns, trends, and evidence gaps. For multiple studies that evaluate similar outcomes using different measurement tools, the findings will be summarized narratively. This approach will allow identification of commonly used outcome measures, methodological heterogeneity, and evidence gaps in assessing the effectiveness of 3D-printed prosthetic rehabilitation across different post-resection maxillofacial defects. Graphical representations, such as evidence maps, bubble plots, or flow diagrams, will be used to summarize the distribution of studies across study designs, interventions, and outcomes.

## Declarations

The authors acknowledge the importance of equity, diversity, and inclusion in research and authorship. This team comprises researchers from diverse academic backgrounds, contributing expertise in oral pathology, oral surgery, prosthodontics, and evidence synthesis. The authors are committed to conducting and disseminating research in a manner that promotes inclusivity, equitable representation, and accessibility of knowledge.

## Conclusion

This scoping review protocol aims to systematically map and synthesize the existing evidence on how post-resection maxillofacial defect characteristics influence the outcomes of 3D-printed prosthetic rehabilitation in oral cancer patients. By examining clinical, functional, aesthetic, and patient-reported outcomes across varying defect types, the review seeks to clarify how anatomical complexity and extent of resection shape rehabilitation success from a prosthodontic perspective. This review will evaluate the scope of 3D-printed prostheses to overcome the challenges and highlight the existing knowledge gaps. The findings are expected to guide clinical decision-making, support the development of personalized rehabilitation strategies, and provide direction for future research aimed at optimising patient-centred care in maxillofacial prosthodontics.

## Supplementary information

Appendices
