# Supplementary material for: Impact of post-resection maxillofacial defects on outcomes of 3D-printed prosthetic rehabilitation in oral cancer patients: a scoping review protocol
Source: BDJ Open. 2026 Aug 14;12:83. doi: 10.1038/s41405-026-00476-3 (PMC13476270; doi:10.1038/s41405-026-00476-3)
Supplement: Supplementary file 1 [file 41405_2026_476_MOESM1_ESM.docx]

**Appendix I: Search strategy**

**Database**: PubMed

**Search conducted on**: 21/07/2026

### **Month/year**: July 2026

**No of records retrieved**: 7

**Filters:** Nil

(("Mouth Neoplasms"[Mesh] OR "oral cancer"[tiab] OR "oral squamous cell carcinoma"[tiab] OR OSCC[tiab] OR "head and neck neoplasms"[Mesh] OR HNSCC[tiab]) AND ("Prosthodontics"[Mesh] OR prosthodontic*[tiab] OR "maxillofacial prosthes*"[tiab] OR obturator*[tiab] OR "dental prosthes*"[tiab] OR "facial prosthes*"[tiab]) AND ("Printing, Three-Dimensional"[Mesh] OR "3D printing"[tiab] OR "additive manufacturing"[tiab] OR "rapid prototyping"[tiab] OR "digital fabrication"[tiab]) AND (defect*[tiab] OR "surgical defect*"[tiab] OR "maxillectomy defect*"[tiab] OR "mandibulectomy defect*"[tiab] OR classification[tiab] OR "defect classification"[tiab] OR "maxillary defect*"[tiab] OR "mandibular defect*"[tiab] OR "palatal defect*"[tiab]) AND ("Treatment Outcome"[Mesh] OR outcome*[tiab] OR "clinical outcome*"[tiab] OR "functional outcome*"[tiab] OR "prosthetic outcome*"[tiab] OR rehabilitation[tiab]))

**Database**: Scopus

**Search conducted on**: 21/07/2026

### **Month/year**: July 2026

**No of records retrieved**: 233

**Filters:** Nil

(("Mouth Neoplasms" OR "oral cancer" OR "oral squamous cell carcinoma" OR OSCC OR "head and neck neoplasms" OR HNSCC) AND (Prosthodontics OR prosthodontic* OR "maxillofacial prosthes*" OR obturator* OR "dental prosthes*" OR "facial prosthes*") AND ("Printing, Three-Dimensional" OR "3D printing" OR "additive manufacturing" OR "rapid prototyping" OR "digital fabrication") AND (defect* OR "surgical defect*" OR "maxillectomy defect*" OR "mandibulectomy defect*" OR classification OR "defect classification" OR "maxillary defect*" OR "mandibular defect*" OR "palatal defect*") AND ("Treatment Outcome" OR outcome* OR "clinical outcome*" OR "functional outcome*" OR "prosthetic outcome*" OR rehabilitation))

**Appendix II: Data extraction form**

# **Section A: Study Characteristics**

| **Author, Year** | **Journal** | **Country** | **Study Design** | **Sample Size (N patients/prostheses)** |
| --- | --- | --- | --- | --- |
|  |  |  |  |  |
|  |  |  |  |  |

# **Section B: Surgical Defect Characteristics**

| **Tumour/ Oral cancer site** | **Defect Classification System Used** | **Type of Defect** | **Defect Extent / Involved Structures** |
| --- | --- | --- | --- |
|  |  |  |  |
|  |  |  |  |

# **Section C: Prosthetic Rehabilitation Details**

| **Type of Prosthesis (obturator/fixed/removable/implant-retained)** | **Fabrication Method (3D-printing technique/material)** | **Design Software / Workflow** | **Follow-up Duration** |
| --- | --- | --- | --- |
|  |  |  |  |
|  |  |  |  |

# **Section D: Outcomes**

| **Outcome Domain** | **Specific Measure** | **Assessment Instrument/Method** | **Type of Defect** |
| --- | --- | --- | --- |
| Functional | Speech |  |  |
|  | Mastication |  |  |
|  | Swallowing |  |  |
|  | Breathing (if applicable) |  |  |
| Prosthetic | Fit and retention |  |  |
|  | Stability |  |  |
|  | Durability |  |  |
|  | Ease of fabrication |  |  |
| Aesthetic | Facial symmetry |  |  |
|  | Patient satisfaction (aesthetic) |  |  |
| Clinical | Complications |  |  |
|  | Prosthesis failure |  |  |
|  | Need for adjustments |  |  |
| Patient-Reported (if reported) | QoL / satisfaction instrument score |  |  |
